# Autoantibodies Targeting AT_1_- and ET_A_-Receptors Link Endothelial Proliferation and Coagulation via Ets-1 Transcription Factor

**DOI:** 10.3390/ijms23010244

**Published:** 2021-12-27

**Authors:** Rusan Catar, Melanie Herse-Naether, Nan Zhu, Philine Wagner, Oskar Wischnewski, Angelika Kusch, Julian Kamhieh-Milz, Andreas Eisenreich, Ursula Rauch, Björn Hegner, Harald Heidecke, Angela Kill, Gabriela Riemekasten, Gunnar Kleinau, Patrick Scheerer, Duska Dragun, Aurelie Philippe

**Affiliations:** 1 Department of Nephrology and Medical Intensive Care, Charité—Universitätsmedizin Berlin, Corporate Member of Freie Universität Berlin and Humboldt-Universität zu Berlin, 10117 Berlin, Germany; melanie.naether@gmail.com (M.H.-N.); znnancy@yeah.net (N.Z.); Philine.wagner@yahoo.com (P.W.); oskar.wischnewski@charite.de (O.W.); angelika.kusch@charite.de (A.K.); bjoern.hegner@gmx.de (B.H.); 2 Center for Cardiovascular Research, Charité—Universitätsmedizin Berlin, Corporate Member of Freie Universität Berlin and Humboldt-Universität zu Berlin, 10117 Berlin, Germany; 3 Shanghai General Hospital, School of Medicine, Shanghai Jiaotong University, Shanghai 200025, China; 4 Berlin Institute of Health, Charité—Universitätsmedizin Berlin, BIH Biomedical Innovation Academy, 10117 Berlin, Germany; 5 Department of Transfusion Medicine, Charité—Universitätsmedizin Berlin, Corporate Member of Freie Universität Berlin and Humboldt-Universität zu Berlin, 10117 Berlin, Germany; julian.milz@charite.de; 6 Department of Cardiology, Charité—Universitätsmedizin Berlin, Corporate Member of Freie Universität Berlin and Humboldt-Universität zu Berlin, 10117 Berlin, Germany; andreas.eisenreich@gmx.de (A.E.); ursula.rauch@charite.de (U.R.); 7 Vitanas Klinik für Geriatrie, 13435 Berlin, Germany; 8 CellTrend GmbH, 14943 Luckenwalde, Germany; heidecke@celltrend.de; 9 Deutsches Rheuma-Forschungszentrum (DRFZ), A. Leibniz Institute, 10117 Berlin, Germany; Angela.Kill@web.de (A.K.); gabriela.riemekasten@uksh.de (G.R.); 10 Department of Rheumatology and Clinical Immunology, CCM, Charité—Universitätsmedizin Berlin, Corporate Member of Freie Universität Berlin and Humboldt-Universität zu Berlin, 10117 Berlin, Germany; 11 Priority Area Asthma & Allergy, Research Center Borstel, Airway Research Center North (ARCN), Members of the German Center for Lung Research (DZL), 23845 Borstel, Germany; 12 Group Protein X-ray Crystallography and Signal Transduction, Institute of Medical Physics and Biophysics, Charité—Universitätsmedizin Berlin, Corporate Member of Freie Universität Berlin and Humboldt-Universität zu Berlin, 10117 Berlin, Germany; gunnar.kleinau@charite.de (G.K.); patrick.scheerer@charite.de (P.S.); 13 DZHK (Deutsches Zentrum für Herz-Kreislauf Forschung), Partner Site Berlin, 13353 Berlin, Germany

**Keywords:** angiotensin, renin–angiotensin system, endothelin-1, systemic sclerosis, renal crisis, autoantibodies, coagulation

## Abstract

Scleroderma renal crisis (SRC) is an acute life-threatening manifestation of systemic sclerosis (SSc) caused by obliterative vasculopathy and thrombotic microangiopathy. Evidence suggests a pathogenic role of immunoglobulin G (IgG) targeting G-protein coupled receptors (GPCR). We therefore dissected SRC-associated vascular obliteration and investigated the specific effects of patient-derived IgG directed against angiotensin II type 1 (AT_1_R) and endothelin-1 type A receptors (ET_A_R) on downstream signaling events and endothelial cell proliferation. SRC-IgG triggered endothelial cell proliferation via activation of the mitogen-activated protein kinase (MAPK) pathway and subsequent activation of the E26 transformation-specific-1 transcription factor (Ets-1). Either AT_1_R or ET_A_R receptor inhibitors/shRNA abrogated endothelial proliferation, confirming receptor activation and Ets-1 signaling involvement. Binding of Ets-1 to the tissue factor (TF) promoter exclusively induced TF. In addition, TF inhibition prevented endothelial cell proliferation. Thus, our data revealed a thus far unknown link between SRC-IgG-induced intracellular signaling, endothelial cell proliferation and active coagulation in the context of obliterative vasculopathy and SRC. Patients’ autoantibodies and their molecular effectors represent new therapeutic targets to address severe vascular complications in SSc.

## 1. Introduction

Systemic sclerosis (SSc) is an autoimmune disease which affects multiple organs, with a wide range of clinical manifestations. Scleroderma renal crisis (SRC), a rare and critical manifestation of SSc, highly affects morbidity and mortality, especially when refractory to treatment [1]. One of its key feature, obliterative vasculopathy, is initiated by vascular remodeling in the interlobar arteries [2], although activation of the coagulation cascade has also been shown to be involved in the pathology [3]. In addition, inhibition of the renin–angiotensin system has dramatically improved the therapy of SRC, although the underlying molecular mechanisms are not well understood [4].

Interestingly, autoantibodies targeting AT_1_R are involved in the occurrence of obliterative vasculopathy in preeclampsia [5], as well as in kidney and heart transplant rejection [6,7,8]. In 2011, their role in the pathophysiology of SSc, as well as their association with renal crisis and increased mortality risk, were identified [9]. Concomitantly, autoantibodies targeting ET_A_R were detected in SSc patients and presented the same features as AT_1_R-IgG. In transplant pathologies, an association with ET_A_R-IgG is already well established [7,10].

In addition, a previous study using receptor-specific IgG isolated from kidney transplant recipients with vascular pathology showed that AT_1_R stimulates TF activation in an acute clinical setting [6]. Passive transfer of patient’s IgG to rats with kidney grafts induced renal lesions [6].

We hypothesized that the endogenous ligands of AT_1_- and ET_A_ receptors activate different intracellular signaling pathways compared to pathological SRC-IgG complexes. The known ability of GPCRs to respond to different agonists such as endogenous ligands, or even antibodies by triggering specific activation of different downstream signaling pathways [11], supports this assumption.

The aim of the current study was therefore to investigate in detail the signaling pathways that are specifically activated by IgG from SRC patients. We demonstrate that SRC-IgG affected the mitogen-activated protein kinase (MAPK)/extracellular signal-regulated kinase 1/2 (ERK1/2) signaling cascade. Moreover, we examined the cellular phenotype triggered by this specific activation to decipher its underlying mechanisms. Our present study provides new insights into the pathogenic molecular mechanisms of obliterative vasculopathy in SRC and the involvement of AT_1_R- and ET_A_R-IgG.

## 2. Results

### 2.1. Autoantibodies against AT_1_R/ET_A_R Activate Ets-1 Transcription Factor via the ERK1/2 Pathway

**Figure 1:**
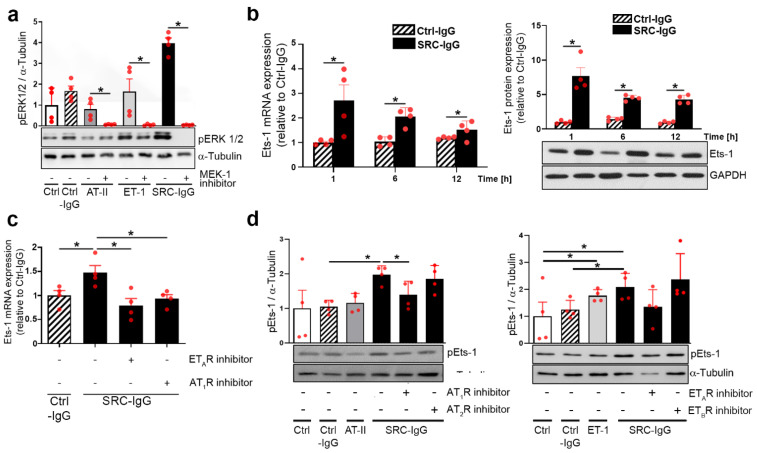
SRC-IgG activate Ets-1. Non-stimulated cells (Ctrl) were used as references when natural ligands were included, whereas Ctrl-IgG served as references when only IgG were used. (**a**) HMEC-1 were stimulated with AT-II, ET-1, Ctrl-IgG or SRC-IgG, with or without pre-incubation with MEK-1 inhibitor. ERK1/2 activation was measured as the pERK/α-Tubulin ratios. (**b**) Ets-1 transcriptional (**left**) and translational levels (**right**) were measured over time after stimulation with Ctrl- or SRC-IgG. (**c**) Specificity was asserted by pre-treatment with an AT_1_R or ET_A_R inhibitor (Valsartan or Sitaxentan, respectively), before stimulation with Ctrl- or SRC-IgG. (**d**) (**left** and **right**) HMEC-1 were incubated with Ctrl-IgG, natural ligands or SRC-IgG with or without pre-incubation with respective receptor blockers. (**a**–**d**) *n* = 4; representative blots are shown. * *p* < 0.05.

We first verified whether ERK1/2 activation in HMEC-1 occurs upon stimulation with SRC-IgG [9]. IgG prepared from SRC patients carrying AT_1_R-/ET_A_R-autoantibodies (IgG levels > 10 U/mL in ELISA) strongly increased ERK1/2 phosphorylation (Figure 1a). This effect was less pronounced with either endogenous ligands AT-II and ET-1 (0.8 and 1.65, respectively, vs. 4.05 with IgG), or IgG from healthy controls (Ctrl-IgG) (Figure 1a).

In agreement with previous results [9], the inhibition of MEK-1 (a kinase upstream from ERK1/2) with the specific inhibitor PD 184,352 abolished ERK1/2 activation by both AT-II and ET-1, as well as by SRC-IgG (Figure 1a). In summary, these assays confirm that ERK1/2 activation in endothelial cells occurs directly after stimulation with SRC-IgG.

Notably, it has been reported that healthy individuals do carry AT_1_R- and ET_A_R-IgG [12]. We demonstrated that although such healthy donor IgG do activate ERK1/2 (Figure 1a), such activation does not involve either AT_1_R or ET_A_R signaling (Figure S1, absence of the effect of AT_1_R and ET_A_R inhibitors on Erk phosphorylation), in contrast to SRC-IgG. 

To determine the intracellular mechanisms underlying ERK1/2 immune activation, we focused further downstream on Ets-1, a transcriptional regulator in AT-II- and ET-1-mediated effects [13,14,15,16], and on the causative TF in autoimmune chronic pathologies [17,18]. Time-dependent HMEC-1 stimulation with SRC-IgG showed a maximal increase in Ets-1 transcript (Figure 1b left) and protein expression (Figure 1b right) one hour after the start of the stimulation, persisting over 12 h in contrast to Ctrl-IgG. Transcriptional SRC-IgG effects were comparable to the effects generated by the stimulation of HMEC-1 with AT-II and ET-1 (Figure S2).

Next, we explored Ets-1 expression caused by SRC autoantibody stimulation by treating endothelial cells with the AT_1_R- or ET_A_R inhibitors Valsartan and Sitaxentan, respectively. Such treatment abolished the increase in Ets-1 at transcriptional level down to 14% with Sitaxentan, and down to 28% with Valsartan (Figure 1c). It is known that ERK1/2 mediates Ets-1 transcriptional activity by phosphorylating threonine 38 (Thr38) of the Ets-1 protein [19]: AT_1_- and ET_A_R inhibitors specifically decreased such Ets-1 phosphorylation (Figure 1d, left and right, respectively). An absence of the effects of AT_2_R and ET_B_R inhibitors or Ctrl-IgG is shown for comparison.

These results demonstrate that SRC-IgG activates ERK1/2-signaling, thereby increasing Ets-1 signaling activity via AT_1_R and ET_A_R.

### 2.2. AT_1_R-/ET_A_R Autoantibodies Trigger Endothelial Cell Proliferation via an ERK1/2—Ets-1 Signaling Pathway

Endogenous ligands induce endothelial cell proliferation via AT_1_R and ET_A_R in endothelial cell types such as human umbilical endothelial vein cells (HUVECs) [20,21,22]; therefore, we next investigated whether SRC-IgG could trigger similar responses in endothelial cells.

**Figure 2:**
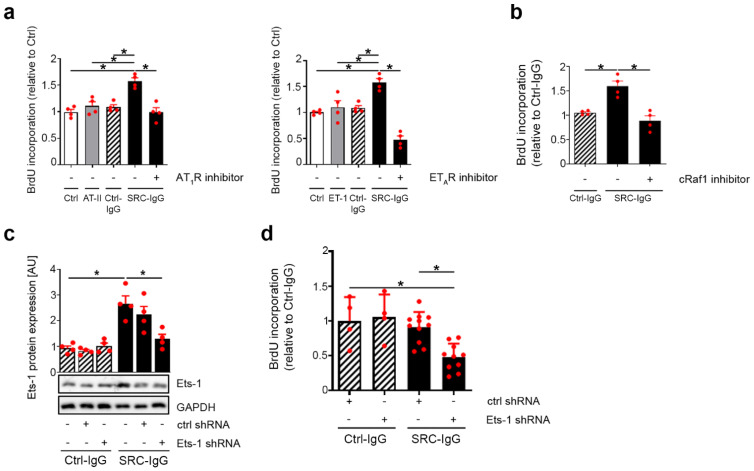
Endothelial cell proliferation elicited by SRC-IgG via ERK1/2–Ets-1 signaling. Non-stimulated cells (Ctrl) were used as reference when natural ligands were included, whereas Ctrl-IgG served as reference when only IgG were used. HMEC-1 were stimulated for 24 h with either natural ligands, Ctrl- or SRC-IgG, and specificity was assessed via two-hour pre-incubation with corresponding receptor inhibitors (**a**) (**left** and **right**) or cRaf1 inhibitor (**b**). (**c**) Abolition of Ets-1 translational regulation by shRNA following six-hour HMEC-1 stimulation. Ctrl shRNA corresponds to a mix of three control shRNA plasmids. Blots were over-exposed to better appreciate the decrease in the protein level. (**d**) Decrease in SRC-IgG induced endothelial cell proliferation by Ets-1 knockdown. (**a**–**c**) *n* = 4, (**d**) 7 ≤ *n* ≤ 11; representative blots are shown. * *p* < 0.05.

BrdU incorporation revealed that 24 h of stimulation with SRC-IgG indeed increased HMEC-1 proliferation (Figure 2a left and right), although endogenous ligands and Ctrl-IgG failed to impact endothelial cell proliferation. Furthermore, pre-incubation with AT_1_- and ET_A_R inhibitors abolished such SRC-IgG-induced proliferative responses, and even decreased it under the control level for Sitaxentan (Figure 2a left and right, respectively), suggesting a receptor-specific proliferative effect of SRC-IgG. Additionally, inhibition of the upstream regulators of ERK1/2 signaling, cRaf1 and MEK-1, prevented this observed proliferation boost (Figure 2b and Figure S3), demonstrating a direct link between activating antibody-mediated stimulation of the ERK1/2 pathway and endothelial cell proliferation in HMEC-1.

It has previously been reported that Ets-1 acts as a negative regulator of endothelial apoptosis during embryogenesis [23]. Therefore, we used shRNA to knock down Ets-1 expression in HMEC-1 before exposing it to healthy or patient IgG. Western blots revealed that shRNA prevented Ets-1 protein level increases in cells stimulated with SRC-IgG but did not change the Ets-1 protein expression in cells exposed to Ctrl-IgG (Figure 2c). This indicates an induction of Ets-1 synthesis in response to SRC-IgG but not Ctrl-IgG. Moreover, shRNA targeting Ets-1 reduced endothelial cell proliferation in cells treated with SRC-IgG but had no effect on cells that received Ctrl-IgG (Figure 2d). 

We conclude that the SRC-IgG-mediated stimulation of AT_1_R/ET_A_R activates ERK1/2, and that its downstream signaling induces the Ets-1 transcription factor which, in turn, promotes endothelial cell proliferation.

### 2.3. Tissue Factor Expression Is Positively Regulated by Ets-1 Binding in the Promoter Region

**Figure 3:**
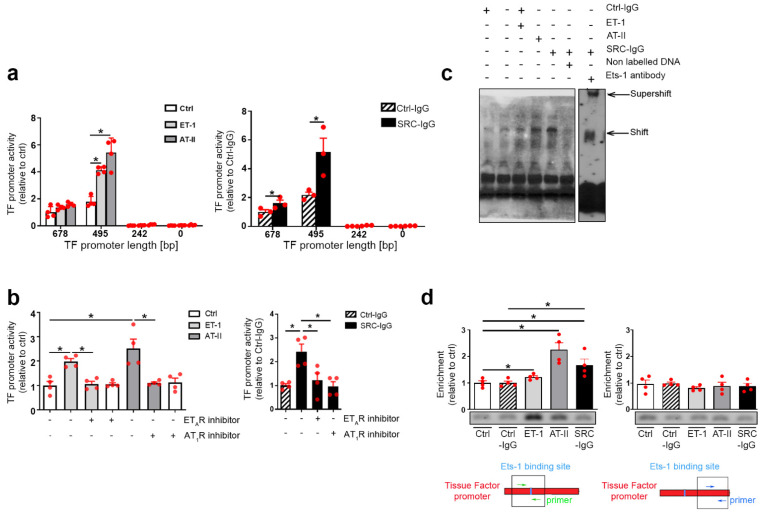
Ets-1 binding to the TF promoter upon AT_1_R/ET_A_R stimulation by either respective natural peptide ligand or in response to SRC-IgG. (**a**) (**left** and **right**) Dual luciferase assay shows a TF promoter activity increase in response to either receptor-activating scenarios as compared with non-stimulated or Ctrl-IgG treated cells. (**b**) (**left** and **right**) Observed activation is abolished by specific AT_1_R or ET_A_R inhibitors. (**c**) EMSA performed with nucleus proteins of endothelial cells incubated with TF promoter DNA. Shift specificity was assessed using non-labeled DNA, the incubation with Ets-1-specific antibodies triggering a supershift. (**d**) (**left** and **right**) Chromatin immunoprecipitation (ChIP) performed using stimulated cells, the DNA of which was precipitated with an antibody directed against Ets-1. (**a**) left, (**b**,**d**) *n* = 4, (**a**) right, (**c**) *n* = 3; representative blots are shown. * *p* < 0.05.

To complete this cascade of Ets-1 regulated mechanisms with effector proteins, we considered TF as a biologically important and potential downstream effector target due to its demonstrated crucial role in the coagulation cascade [24,25]. To establish a direct link between Ets-1 and TF regulation in SRC-related vascular obliteration, we performed promoter analysis of the TF gene using dual luciferase assays. TF promoter deletion constructs showed that a motif located within 495 base pairs downstream of TF transcription initiation site is essential for TF expression induced by both endogenous ligands (Figure 3a left) and SRC-IgG (Figure 3a right). AT_1_R- and ET_A_R-inhibitors abolished all increases in promoter activity, again demonstrating specificity to these receptors (Figure 3b, left and right).

The luciferase assays were expanded to the endogenous TF promoter. Hence, we first assessed the presence of an active Ets-1 binding site in the promoter. An EMSA (electrophoretic mobility shift assay) showed specific binding of Ets-1 to the TF promoter, which was even higher upon SRC-IgG stimulation. This binding was lost upon the addition of specific non-labeled DNA. Moreover, addition of the Ets-1 antibody confirmed that the DNA fragment was occupied by Ets-1 (Figure 3c). We performed chromatin immunoprecipitation (ChIP) with anti-Ets-1 antibodies on stimulated HMEC-1 (Figure 3d left (Ets-1 binding site flanking primers) and right (binding site-independent primers, negative control)) and confirmed our promoter analyses conclusions (Figure 3a); immunoprecipitation with Ets-1 antibodies specifically yielded a Ets-1 binding site PCR product of expected size, enriched upon ligand or activating IgG stimulation.

Taken together, these findings demonstrate that an active Ets-1 binding site exists in the TF promoter, >495 base pair upstream of transcription initiation site, which stimulates TF expression in HMEC-1 in response to SRC-IgG.

### 2.4. SRC-IgG-Mediated Ets-1 Signaling Induces TF-Dependent Proliferation

**Figure 4:**
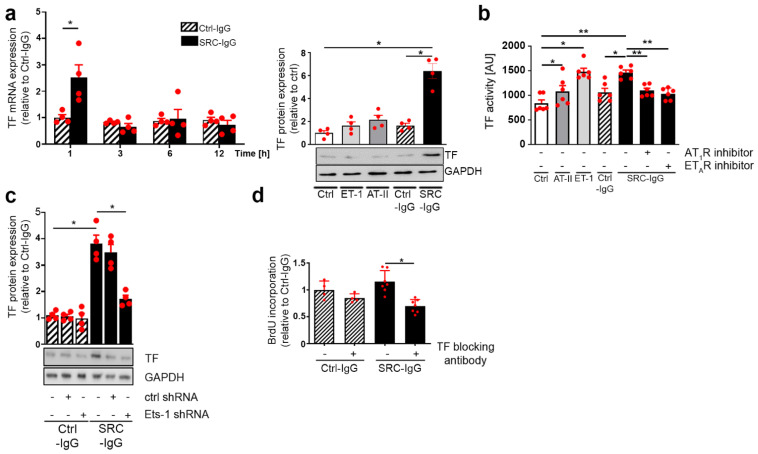
TF involvement in endothelial cell proliferation. Non-stimulated cells (Ctrl) were used as a reference when natural ligands were included, whereas Ctrl-IgG served as a reference when only IgG were used. (**a**) Transcriptional (**left**) and translational analysis (**right**) of TF after endothelial cell stimulation. (**b**) Specific inhibition of AT_1_R/ET_A_R abolishes TF activity increase. (**c**) Ets-1 knockdown abolishes TF protein synthesis. (**d**) BrdU incorporation shows that pre-incubation with a TF-blocking antibody annihilates endothelial cell proliferation elicited by SRC-IgG. (**a**,**c**) *n* = 4, (**d**), 4 ≤ *n* ≤ 7; representative blots are shown. * *p* < 0.05, ** *p* < 0.01.

After confirming that AT_1_R/ET_A_R stimulation increased Ets-1 binding to the TF promoter (Figure 3), we further investigated the effect on TF mRNA in HMEC-1. Transcriptional levels of TF transiently tripled one hour following SRC-IgG (but not Ctrl-IgG stimulation; Figure 4a left), which, in turn, became considerably upregulated TF protein levels after six hours of stimulation (Figure 4a right, endogenous ligands and Ctrl-IgG had minimal effects).

We further tested whether increasing TF expression modulated its activity. AT-II and ET-1 stimulation indeed increased TF activity (46% and 87% increases in comparison to the control, respectively, Figure 4b) [26], whereas stimulation with activating IgG exhibited a potential comparable to ET-1 (79% increase compared to control, Figure 4b). Ctrl-IgG had an effect in the range of what was observed with AT-II. The receptor specificity was again confirmed by the lack of increase in TF activity upon pre-treatment with AT_1_/ET_A_R inhibitors (Figure 4b). Similarly, Ets-1 knockdown in HMEC-1 demonstrated the essential role of Ets-1 for TF activity induced by SRC-IgG, whereas Ets-1 was not involved with Ctrl-IgG (Figure 4c). This was corroborated by supplemental data, where SRC-IgG, but not Ctrl-IgG, stimulated thrombin protein release in HMEC-1 (Figure S4).

Finally, we investigated the link between TF and endothelial cell proliferation. For this purpose, HMEC-1 were pre-incubated with specific TF-inhibiting antibodies before stimulation with Ctrl- or SRC-IgG. TF-inhibiting antibodies specifically and significantly reduced SRC-IgG-induced endothelial cell proliferation (39% decrease), whereas they only had a limited effect upon stimulation with Ctrl-IgG (15% decrease) (Figure 4d).

**Figure 5:**
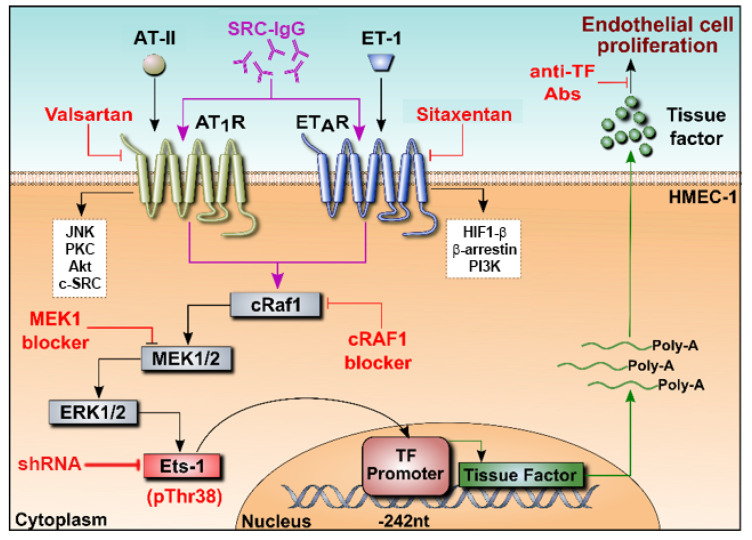
Proposed intracellular cascade following AT_1_R and ET_A_R activation by SRC-IgG. Binding of SRC-IgG to the receptors triggers the activation of cRaf1, MEK, ERK1/2 and, in turn, of Ets-1, through phosphorylation of its Thr38. Once activated, Ets-1 binds to the promoter of TF, triggering its expression (mRNA and protein). This intracellular pathway results in endothelial cell proliferation, inducing obliterative vasculopathy in SSc patients.

These results clearly demonstrate that TF acts as a downstream effector of Ets-1 in the ERK 1/2 immune-induced signaling pathway mediating endothelial cell proliferation in SRC (Figure 5).

## 3. Discussion

Our study provides evidence that activating autoantibodies directed against AT_1_R/ET_A_R trigger a specific signaling pathway linking the transcriptional control of endothelial cell proliferation with increased pro-coagulatory properties in scleroderma renal crisis (SRC). Exposure of microvascular endothelial cells to receptor-activating antibodies triggered ERK1/2 phosphorylation, increased further downstream Ets-1 transcription factor activation and led to subsequent synthesis of TF as Ets-1 target gene and initiator of coagulation. This cascade was not observed upon activation with the natural ligands of the receptors. With this newly described molecular mechanism, we expand our previous findings [9] and offer potential explanations as to why SRC patients harboring AT_1_R- and ET_A_R-IgG present an increased risk for the earlier occurrence of severe and potentially lethal vascular complications in the kidney.

In this study, we concentrated on SRC-IgG and their involvement in the pathogenesis of SRC. Antibodies other than AT_1_R- and ET_A_R-IgG have been associated with SRC. Anti-RNA polymerase III antibodies (ARA), especially, have been associated with kidney manifestations of systemic sclerosis [27,28,29]. However, although these antibodies constitute a biomarker of SRC, no studies show that these antibodies participate actively in the occurrence of the disease. In the publication from Mouthon L. et al., three out of the four SRC patients tested presented anti-Topoisomerase I antibodies (ATA) [27]; according to the literature, antinuclear antibodies, among which include ARA and ATA, are mutually exclusive [30]. Moreover, ATA is associated with the occurrence of interstitial lung disease, but not with SRC [31].

Our work presents the involvement of the ERK1/2–Ets-1 signaling pathway in the occurrence of SRC vascular lesions. Members of the ETS transcription factor family participate in the regulation of inflammatory and angiogenic responses in endothelial cells [19]. The Ets-1 protein structure contains a conserved DNA-binding domain, forming a winged helix-turn-helix structure. Other Ets-1 target genes, such as the IL-8 gene, can be induced by SRC-IgG stimulation, as we have previously demonstrated [32]. Ets-1 activation is also involved in chronical autoimmune pathologies such as rheumatoid arthritis [18] and lupus [17].

Several reports indicate a role of Ets-1 in endothelial cell proliferation [33,34]. In a pathological paradigm of murine carotid–jugular fistula, Ets-1 expression has been shown to be increased in the neointima and overlying endothelium [35]. In terms of signaling, chronic infusion of AT-II in mice induces Ets-1 expression in endothelial cells [16]. Our results imply that SRC-IgG cause endothelial proliferation through Ets-1-mediated transcriptional program, notably its subsequent chronic activation of TF. TFs act as the primary initiator of the in vivo coagulation cascade. The endothelium itself has been shown as an important source of TF [36]. Uncontrolled endothelial proliferation, combined with increased pro-coagulatory properties of the endothelium of middle-sized arteries and arterioles, can contribute to the formation of onion-skinning concentric narrowing, which then leads to the obliterative vasculopathy observed in kidneys during SSc [37]. Studies of antiphospholipid syndrome have demonstrated that autoantibodies can exert an influence on TF expression on either monocytes [38] or endothelial cells [39].

Notably, the differences observed between the proliferation measures in Figure 3a,b,d are most likely due to the use of distinct experimental methods (namely, a Roche proliferation kit for the 3a/b and BrdU immunofluorescence for 3d), not actual biological difference.

Activation of the ERK–Ets-1–TF axis was investigated here in human endothelial cells. However, AT_1_R and ET_A_R are also strongly expressed in renal vascular smooth muscle cells, (vSMCs) [40,41]. Moreover, SRC has been associated with the proliferation of both endothelial and vascular smooth muscle cells [42]. Hence, we cannot exclude that the signaling pathway we describe also occurs in vSMCs, and further studies are needed to clarify whether these cells are also affected by SRC-IgG. One such modern approach to identify how SRC-IgG differentially influence EC and vSMC would be single-cell sequencing. Two recent articles have thus investigated the relative impact of EC and vSMC in hypertension-induced vascular remodeling [43,44]. In a mouse model of salt-induced hypertension, Zhang et al. showed that hypertensive vessels present increased smooth muscle cell populations, partly due to endothelial-to-mesenchymal cell transition [44]. In a context of systemic sclerosis, single-cell sequencing was used to study immune cell heterogeneity between patients and healthy controls using skin samples [45], or whole endothelial cell genetic patterns in individuals with or without the disease [46]. Such investigations would be of particular interest in blood vessels from SRC patients to identify the consequences of SRC-IgG on specific cell types. Obviously, a single-cell sequencing approach in blood vessel would require spatial cell type identification, one of which possibilities could be to couple this method with spatial transcriptomics, as the group of H. Benjamin published recently for acute kidney injury [47].

We observed differences between the actions mediated by SRC-IgG and natural ligands. In HMEC-1, patient IgG and ET-1 activated ERK 1/2 and Ets-1, although ET-1-driven activation was significantly lower in comparison with SRC-IgG. In the literature, the ET-1 activation of ERK 1/2–Ets-1 signaling has exclusively been reported once in human peritoneal mesothelial cells [33]. In contrast, AT-II has been reported three times as an Ets-1 activator, but exclusively in renal or cardiac fibroblasts [48,49,50].

In addition, in contrast to SRC-IgG, neither AT-II nor ET-1 increased HMEC-1 proliferation. AT-II has, however, recently been associated with increased proliferation in lymphatic endothelial cells, and previously in human umbilical vein endothelial cells (HUVECs) [20,51]. AT_1_R stimulation triggered angiogenesis in both instances, a mechanism not involved in the present work. Additionally, three reports demonstrated that ET-1 increases endothelial cell proliferation [52,53,54], although in each case, the ET_A_R inhibitor was only mildly efficient, whereas the ET_B_R inhibitor blocked the observed effects.

Finally, TF protein expression was only moderately increased in response to either natural ligand (in contrast to SRC-IgG), which was mirrored by a moderate induction of TF activity compared with SRC-IgG. AT-II-induced increases in TF expression had already been reported in monocytes [55] and transgenic rats showing cardiac vasculopathy [56]. ET-1 links to TF are less well documented, but have been shown in children with bronchopulmonary dysplasia [57], whereas the induction of TF by AT_1_R-IgG has already been reported in women with preeclampsia and in acute kidney graft rejection by our group [6,58]. In the present study, TF activation by SRC-IgG was mediated by Ets-1. On the other hand, our previous sister reports in preeclampsia and kidney graft rejection involved AP-1 and/or NF-κB [6,58]. Articles establishing TF activation by AT-II also involved NF-κB [55,56]. Hence, although the massively decreased TF expression following Ets-1 inhibition makes such a hypothesis unlikely, further studies are needed to establish whether AP-1 and NF-κB also participate in SRC and TF induction.

According to the data presented here, AT_1_R inhibitors should prove beneficial in the treatment of SRC. Actually, several reports about patients treated with angiotensin receptor blockers (ARBs) exist, but the general picture remains controversial: in 1997, an article reported the failure of losartan to control SRC in a patient [59], whereas another case report published in 2005 described a resolution of the crisis under treatment with ARB [60]. Most patients presenting SRC receive angiotensin-converting enzyme inhibitors (ACEi), a treatment which leads to a significant increase in the 5-year survival of SRC patients [61]. Nevertheless, a recent two-year prospective survey demonstrated that exposition to ACEi prior to the onset of SRC was associated with a higher risk of death, even when pre-existing hypertension backgrounds were taken into account. In contrast, ARB did not present these negative effects [4]. Moreover, analysis of the cohort from the European Scleroderma Trial and Research group (EUSTAR) showed that the cumulated incidence of SRC was higher for patients treated with ACEi, whereas ARBs had no influence [62]. Finally, another recent study revealed that ARB could delay the development of major vascular complications, such as SRC or pulmonary arterial hypertension in SSc patients, whereas use of ACEi was rather associated with an earlier onset of such complications [63]. These data underline the need for further large-scale, comparative studies to determine the effectiveness of ARB in the treatment of SRC. Taking into account our results on SRC-IgG in the pathogenesis of SRC mediated by AT_1_- and ET_A_R, the use of ARBs appears more likely to be beneficial compared with ACEi, as mostly reported.

### 3.1. Study Limitations

We are aware that our study has limitations. Our IgG preparations stemmed from four patients with scleroderma renal crisis. However, as emphasized in a recent article, this complication has become increasingly rare over the years [64].

As a second limitation, we did not investigate the activation of NF-κB and AP-1 transcription factors that have already been associated with AT_1_R-Abs in acute kidney graft rejection. Nonetheless, the considerable effect of Ets-1 shRNA-mediated knockdown on proliferation and TF expression leads us to the conclusion that Ets-1 is a major player in the signaling axis that could lead to the endothelial phenotype of SRC.

Moreover, the spectrum methods is constantly evolving, and single-cell sequencing has noticeably changed the way studies are conducted. Applying this method to our experimental setting could hold the key to understand how SRC-IgG are affecting differentially specific blood vessels cell types.

Finally, IgG isolated from healthy controls do induce ERK1/2 activation and endothelial cell proliferation, but without involving AT_1_R, ET_A_R or Ets-1. Future studies should assess the exact particularities of patients and healthy autoantibodies.

### 3.2. Conclusions and Perspectives

Our results highlight a new mode of signaling and transcriptional regulation of TF by Ets-1, induced by autoantibodies against AT_1_R and ET_A_R. This adds a new layer of complexity to the concept, in which endothelial injury can be separated into two stages of response: first, a rapid, initial reaction; and second, a slower phenotypic response, the latter of which could trigger vascular remodeling [65]. Our studies further point to additional molecular mechanisms involving Ets-1 and TF. Hence, Ets-1 has been involved in dermal and renal fibrosis, and targeting this factor showed improvements in collagen dysregulation [13,66]. Concomitantly, TF has been associated with lung fibrosis, and therapeutic interventions with Dabigatran (anticoagulant) were conclusive [67]. Therapeutic strategies in SRC could be improved, associating specific targeting of new actors and known beneficial treatments, such as plasmapheresis [68].

## 4. Materials and Methods

### 4.1. Clinical Samples and IgG Isolation

Serum and plasma were obtained from four patients treated for angiotensin-converting enzyme I (ACEI) inhibitor-refractory SRC in our clinic between January 2006 and October 2010, after written informed consent and local ethics committee approval (EA1/013/705) had been received. SRC was defined by an otherwise unexplained rapid decline in renal function (increase in serum creatinine ≥50%) in patients with SSc. Diagnosis was confirmed by renal biopsy showing obliterative vasculopathy of arteries and arterioles in all cases (Table S4). All healthy and SRC individuals were tested for the presence of AT_1_R and ET_A_R antibodies using a sandwich ELISA (CellTrend GmbH, Luckenwalde, Germany), as described in detail in [69]. Only SRC patients showed high (above 10 U/mL) AT_1_R- and ET_A_R-IgG levels. All experiments were performed with four different individual IgG preparations isolated from patient plasma. IgG were isolated with HiTrap Protein G columns (GE Healthcare, Chicago, IL, USA). Briefly, plasma originating from the first plasmapheresis was aliquoted in 50 mL samples and frozen. Aliquots (50 mL) were thawed upon experimentation, mixed 1:1 with binding buffer (0.02 M Na_2_HPO_4_, pH 7.0) and filtered through a 0.45 µm filter to eliminate debris. The mix was then passed twice through a protein G column to bind IgG. Unspecific binding was removed by washing the column with binding buffer. IgG were eluted using a low-pH elution buffer (0.1 M Glycin-HCl, pH 2.7), and harvested in fractions 4 to 8. The elution buffer was then neutralized with 1 M Tris, pH 9.0, and IgG were dialyzed against low-glucose DMEM overnight. 

### 4.2. Cell Culture, Stimulation and Transfection

Human dermal microvascular endothelial cells (HMEC-1) were cultured in endothelial cell growth medium (PAA Laboratories GmbH, Pasching, Austria) with 5% (*v/v*) FBS. These cells reliably express AT_1_R and ET_A_R. For stimulation experiments, HMEC-1 were serum-starved for 24 h, then stimulated with SRC-IgG (1.5 mg/mL), ET-1 (0.1 µmol/L), AT-II (1 µmol/L). Pre-incubation with AT_1_R or ET_A_R inhibitors (Valsartan, Sigma Aldrich, Saint Louis, MI, USA and Sitaxentan, Pfizer, New York, NY, USA, respectively), MEK or Raf inhibitors (PD 184,352, Axon Medchem, Reston, VA, USA and GW5074, Sigma Aldrich, Saint Louis, MI, USA, respectively) were performed for two hours, whereas TF-blocking antibody (clone 5G9) was pre-incubated for 15 min. Western blots were performed as described previously [9]. Cells were rinsed twice with ice-cold 10 mM HEPES, 150 mM NaCl buffer, pH 7.5, before lysis in buffer containing 40 mM Tris/HCl, pH 8.0, 4 mM EDTA, 20% glycerol, 276 mM NaCl, 2% Triton X-100, 1 mM sodium vanadate, 2 mM sodium pyrophosphate, 10 mM sodium fluoride, 10 mM β-glycerophosphate, and complete protease inhibitor cocktail (Roche Diagnostics, Switzerland). After 20 min incubation on ice, the lysates were cleared by centrifugation at 14,000 rpm at 4 °C for 15 min. Protein concentrations were determined with Bio-Rad Protein Assay. Aliquots with a 40 μg total protein content were boiled in SDS-Laemmli buffer with 100 mM DTT for 5 min. Samples were loaded onto 10% Bis-Tris polyacrylamide gels and separated by electrophoresis. Proteins were transferred onto nitrocellulose membranes (GE Healthcare, Sweden), which were then blocked for one hour at room temperature with 5% non-fat milk (Applichem, Germany) and 1% bovine serum albumin (SERVA, Germany) in 0.1% Tween-Tris-buffered saline. Membranes were probed with phospho-ERK (Cell Signaling, Danvers, MA, USA), α-Tubulin (Sigma Aldrich, Saint Louis, MI, USA), Glyceraldehyde 3-phosphate dehydrogenase (GAPDH) (Santa Cruz, Dallas, TX, USA or Hytest Ltd., Turku, Finland), p38-Ets-1 (Invitrogen, Carlsbad, CA, USA), Ets-1 (Santa Cruz, Dallas, TX, USA), TF (American Diagnostica, Pfungstadt, Germany) antibodies. shRNA vectors provided by Santa Cruz were used to deliver scrambled or Ets-1 shRNA. Cells were transfected with TurboFect (Fermentas, Waltham, MA, USA), following the manufacturer’s instructions. After three hours, the medium was changed to starvation medium. On the next day, cells were stimulated.

### 4.3. Proliferation Assays

Proliferation was measured by Bromodeoxyuridine (BrdU) assay using either a kit (Roche Diagnostics, Switzerland) (Figure 3a,b) or immunofluorescence with anti-BrdU antibody from Cell Signaling (Danvers, MA, USA) or Alexa Fluor 488 mouse anti-BrdU antibody from BD Pharmingen (San Diego, CA, USA) (Figure 3d and Figure 5d), as previously described [70]. Briefly, cells were seeded on glass coverslips coated with 0.2% porcine gelatin. After starvation overnight, cells were stimulated. One hour before the end of the stimulation, BrdU was diluted to a final concentration of 20 µM in the cell culture medium. Cells were then washed and fixed with paraformaldehyde 4% for 15 min. Permeabilization was performed with 0.5% Triton X100 for 3 min and cells were finally blocked overnight at 4 °C in BSA 1%. On the next day, the BrdU antibody was diluted 1/1000 in a PBS solution containing 1% BSA, 33 mM Tris-HCl pH 8.0, 0.33 mM MgCl_2_ and 0.5 mM Mercaptoethanol and Dnase I 2 U/µL for one hour at 37 °C. After washing, secondary fluorescent antibody was incubated for 1 h 30 at 37 °C. DAPI was used to counterlabel nuclei. Cell counting was performed automatically using ImageJ version 1.48.

### 4.4. RNA Extraction and Quantitative RT-PCR

All primer sequences are provided in the Supplementary Material (Table S1). Total RNA was extracted from cultured cells, and quantitative reverse transcription (RT)-PCR was performed with an Applied Biosystems 7500 Fast Real-Time PCR system (Applied Biosystem, Waltham, MA, USA) using Power SYBR Green PCR Master Mix. Relative amounts of gene transcript were calculated by the cycle threshold method and normalized for the endogenous reference (β2-microglobulin).

### 4.5. Reporter Constructs and Luciferase Assay

All primer sequences are provided in the Supplementary Material (Table S2). PCR products were cloned into a luciferase vector (pGL4.10, Promega, Madison, WI, USA). HMEC-1 were transfected with the TF reporter plasmid and co-transfected with the reference pRL-TK renilla plasmid. Luciferase activity was assessed with the dual-luciferase reporter assay system (Promega, Madison, WI, USA).

### 4.6. Nuclear Extracts and Electrophoretic Mobility Shift Assay (EMSA)

All primer sequences are provided in the Supplementary Material. Oligonucleotide probes were labeled using a Biotin 3’ End DNA Labeling Kit (Thermo Scientific, Darmstadt, Germany). Nuclear extracts were prepared using an NE-PER Nuclear and Cytoplasmic Extraction Kit (Thermo Scientific, Waltham, MA, USA). The probe for Ets-1 used in EMSA (5′-TGGGCAAAGCATCCGGGAAATGCC-3′) corresponds to the TF promoter region: −498 to −475 bp. The binding mixture contained 5 µg nuclear extract, 20 fmol labeled double-stranded probe, 1 µg poly-dI/dC, and 1X reaction buffer. Incubation was performed at room temperature for 30 min. Protein–DNA complexes were then analyzed by electrophoresis in 6% non-denaturing polyacrylamide gels and visualized using a LightShift Chemiluminescent EMSA Kit (Thermo Scientific, Darmstadt, Germany). In supershift experiments, nuclear extracts were incubated with Ets-1 antibody (Abcam, Cambridge, UK) before adding the biotin-labeled probe.

### 4.7. Chromatin Immunoprecipitation Assay (ChIP)

Formaldehyde cross-linking and ChIP were performed using the ChIP-IT High Sensitivity Kit (Active Motif, Carlsbad, CA, USA), following the manufacturer’s instructions. Briefly, after test exposure, HMEC-1 were fixed for 10 min with complete cell fixation solution containing 37% formaldehyde, and sonicated to generate 500–800 bp DNA fragments. Immunoprecipitation was performed with protein G agarose beads and 4 μg of Ets-1 antibody (Abcam, Cambridge, UK). Chromatin extracts were incubated with the antibodies at 4 °C overnight under mild shaking, and ChIP DNA was eluted according to the manufacturer’s instructions. Precipitated DNA was purified and amplified by PCR with specific TF primers positive or negative for the presence of the Ets-1 binding site. PCR amplifications were performed with the primers listed in the Supplementary Material (Table S3).

### 4.8. Chromogenic TF Activity Assay and Thrombin Secretion

TF activity was measured as described previously [71]. Stimulated or non-stimulated HMEC-1 were washed twice with ice-cold PBS. Cells were incubated for 15 min at 37 °C with 0.1 M n-octyl-β-D-glucopyranoside in HEPES buffer (200 μL total). TF activity was measured by adding 100 μL of the sample to a solution of 2 nM factor (F)VIIa, 150 nM FX, and 5 mM CaCl_2_. Chromogenic FXa substrate (American Diagnostica, Pfungstadt, Germany) was added to each well (0.5 mM final). At intervals, samples were transferred to a microtiter plate containing EDTA buffer, which terminated the generation of FXa. OD increments were measured at 405 nm for 30 min using a kinetic ELISA plate reader (37 °C, Molecular Devices, San Jose, CA, USA). TF activity units were assessed by a standard curve. Thrombin secretion was measured with an AssayMax Human Thrombin ELISA Kit (Assaypro LLC, St Charles, MO, USA), as described previously [72].

### 4.9. Statistics

All statistical analyses were performed with GraphPad Prism v8.00 (GraphPad Software, San Diego, CA, USA). Statistical significance was assessed using Mann–Whitney U tests (* *p* < 0.05, ** *p* < 0.01, *** *p* < 0.001). Data are presented as the mean of independent experiments with the amount of individual experiments provided in figure legends as “*n* = ”; error bars depict the SEM calculated from these independent experiments. All experiments were performed with individual patient IgG and the results were only pooled for the graphical representation and statistical analysis.

## Data Availability

The data presented in this study are available on request from the corresponding author. The data are not publicly available as they may be used for further study in group.

## Supplementary Materials

The following are available online https://www.mdpi.com/article/10.3390/ijms23010244/s1.
