# Supplementary material for: Autoantibodies Targeting AT1- and ETA-Receptors Link Endothelial Proliferation and Coagulation via Ets-1 Transcription Factor
Source: Int J Mol Sci. 2021 Dec 27;23(1):244. doi: 10.3390/ijms23010244 (PMC8745726; doi:10.3390/ijms23010244)
Supplement: Supplementary file 1 [file ijms-23-00244-s001.zip › ijms-1499795-supplementary.pdf]

Online supplement

Supplementary Material and Methods	Page 1 to 3
Supplementary Figures	Page 4 to 5
Supplementary Table S1	Page 2
Supplementary Table S2	Page 2
Supplementary Table S3	Page 3
Supplementary Table S4	Page 6

Supplementary Material and Methods

RNA extraction and real-time RT-PCR

Primer sequences are reported in suppl. Table 1, all shown as 5’-3’.

**Ets-1 GenBank NM_001143820.1**	**Forward primer**	ACCCAGCCTATCCAGAATCC
	**Reverse primer**	ATGAAGCTGGGCTCTGAGAA
**Tissue Factor GenBank NM_001993.4**	**Forward primer**	TGATGTGGATAAAGGAGAAAACTACTGT
	**Reverse primer**	CTACCGGGCTGTCTGTACTCTTC

Table S1 : ***Primer sequences for real-time PCR.***

Reporter constructs and Luciferase assay

Human genomic DNA was amplified by PCR using a common reverse primer complementary to the DNA sequence 178 base pairs downstream of the translation start site in TF gene *F3* (Suppl. Table 2) and forward primers located -678, -495 or -242 bp upstream of the translation start site (Suppl. Table 2), all shown as 5’-3’.

F3	**Forward primer [-678]**	TGATCAGGTACCGAGCCAACTGACCCTCAGAC
	**Forward primer [-495]**	TGATCAGGTACCACGTTTACTTCGCTGCAGGT
	**Forward primer [-242]**	TGATCAGGTACCGACCCGGGCAACTAGACC
	**Reverse primer**	CAGGCAGAGCTCGCAGGGGTCTCCATGTCTAC

Table S2 : ***Primer sequences for promoter luciferase reporter assays.***

Nuclear extracts and Electrophoretic Mobility Shift Assay (EMSA)

For EMSA, we used an Ets-1 [5’-TGGGCAAAGCATCCGGGAAATGCC-3’] probe (-475 to -498 from translation start).

Chromatin immunoprecipitation assay (ChIP)

Primer sequences listed in suppl. Table 3 were used, all shown as 5’-3’.

Specific Tissue Factor primers	Positive	**Forward primer**	AGAGGCAAACTGCCAGATGT
		**Reverse primer**	TGTCTACCAGTTGGCGGAGG
	Negative	**Forward primer**	GAATCACATCCCAGGTGGAG
		**Reverse primer**	GAAGCAGAAAGTTGCCCTTG

Table S3: ***Primer sequences for CHIP.***

Supplementary figures

Suppl. Figure S1

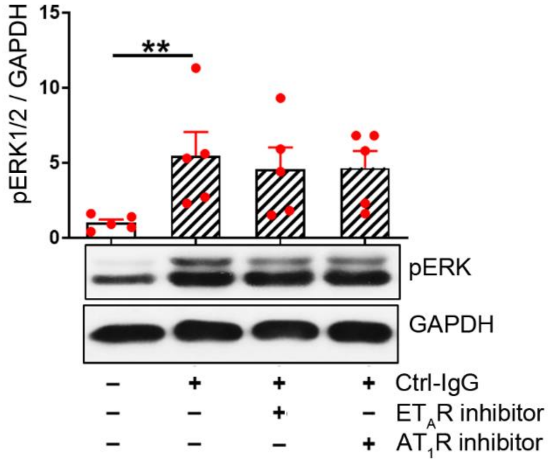

Legend Figure S1. ERK1/2 activation (phosphorylation) after stimulation with IgG isolated from healthy individuals (Ctrl-IgG). *HMEC-1 were stimulated 15 minutes with Ctrl-IgG and specificity was assessed via one-hour pre-incubation* AT_1R *or* ET_AR *inhibitors (Valsartan and Sitaxsentan, respectively). Data derived from five experiments.*

Suppl. Figure S2

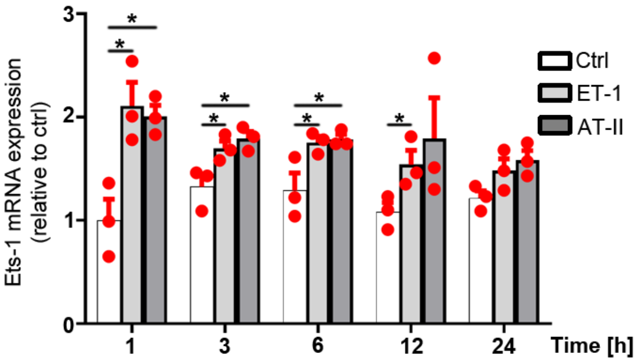

Legend Figure S2. Transcriptional regulation of Ets-1 after stimulation with Endothelin-1 (ET-1) or Angiotensin II (AT-II). *Non-stimulated cells were used as control. HMEC-1 were stimulated for increasing time. Data derived from three experiments.*

Suppl. Figure S3

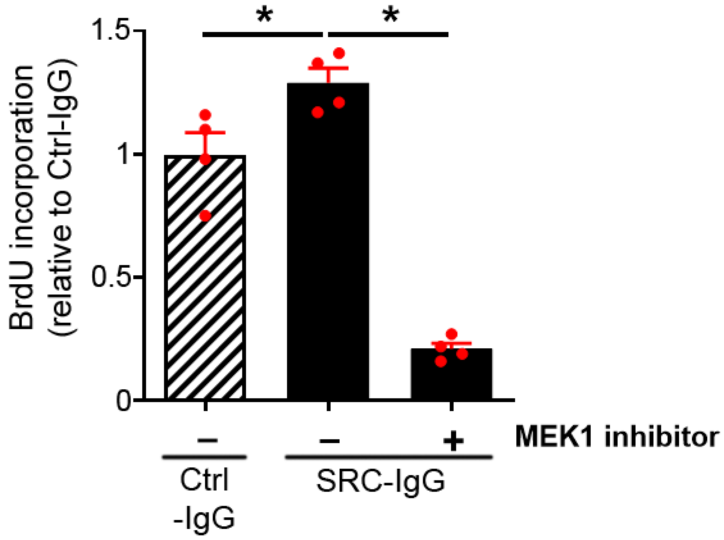

***Legend Figure S3. Endothelial cell proliferation triggered by SRC-IgG via ERK1/2 – Ets-1 signalling.** Ctrl-IgG-stimulated cells were used as control. HMEC-1 were stimulated 24 hours and specificity was assessed via two-hour pre-incubation MEK1 inhibitor. Data derived from four experiments.*

Suppl. Figure S4

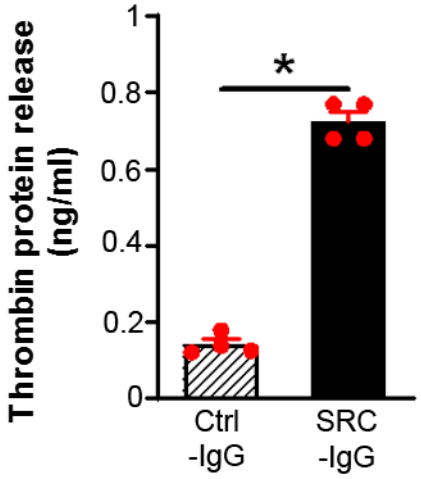

***Legend Figure S4. Thrombin protein release after stimulation with Ctrl- or SRC-IgG.** HMEC-1 were stimulated 24 hours. Data derived from four experiments.*

	Patient no.	1	2	3	4	median (min-max)<br>frequency (%)
**Patient characteristics**	**age [years]**	47	52	47	68	49.5 (47-68)
	**sex**	f	f	f	f	4/4 (100%) female
	**disease duration [month]**	91	91	70	5	80.5 (5-91)
	**diffuse/limited SSc**	limited	diffuse	diffuse	diffuse	3/4 (75%) diffuse
	**antibodies**	-	Scl-70	Scl-70	Scl-70	3/4 (75%) Scl-70 pos.
	**mean AT_1R-IgG level [U/mL]**	16.94	23.77	29.89	15.45	20.36 (15.45-29.89)
	**mean ET_AR-IgG level [U/mL]**	17.00	28.15	32.14	15.96	22.58 (15.96-32.14)
**Clinical presentation**	**hypertensive (≥140/85 mmHg)**	+	-	+	-	2/4 (50%)
	**systolic [mmHg]**	>300	90	220	120	170 (90->300)
	**diastolic [mmHg]**	140	60	100	85	92.5 (60-140)
	**initial kidney function/creatinine [mg/dL]**	3.64	4.68	anuria, HD	4.00	1/4 (25%) HD; 4.00 (3.64-4.68)
**Histology**	**preglomerular TMA**	+	+	-	-	2/4 (50%)
	**glomerular TMA**	+	+	-	-	2/4 (50%)
	**fibrinoid necrosis**	+	+	-	-	2/4 (50%)
	**myxoid intimal deposition**	+	+	+	-	3/4 (75%)
	**concentric intimal sclerosis**	+	+	+	+	4/4 (100%)
	**IF/TA**	20%	20%	<5%	15%	3/4 (75%)

Table S4: ***Demographical characteristics.***

f - female; Scl-70 – anti-topoisomerase I; TMA - thrombotic microangiopathy; IF/TA - interstitial fibrosis/tubular atrophy
